# CRISPR/Cas Technology in Pig-to-Human Xenotransplantation Research

**DOI:** 10.3390/ijms22063196

**Published:** 2021-03-21

**Authors:** Natalia Ryczek, Magdalena Hryhorowicz, Joanna Zeyland, Daniel Lipiński, Ryszard Słomski

**Affiliations:** 1 Institute of Human Biology and Evolution, Adam Mickiewicz University in Poznan, Uniwersytetu Poznańskiego 6, 61-614 Poznan, Poland; 2 Department of Biochemistry and Biotechnology, Poznan University of Life Sciences, Dojazd 11, 60-632 Poznań, Poland; magdalena.hryhorowicz@gmail.com (M.H.); jzeyland@gmail.com (J.Z.); lipinskidaniel71@gmail.com (D.L.); 3 Institute of Human Genetics, Polish Academy of Sciences, Strzeszyńska 32, 60-479 Poznań, Poland; slomski@up.poznan.pl

**Keywords:** CRISPR/Cas, genetic engineering, xenotransplantation

## Abstract

CRISPR/Cas (clustered regularly interspaced short palindromic repeats linked to Cas nuclease) technology has revolutionized many aspects of genetic engineering research. Thanks to it, it became possible to study the functions and mechanisms of biology with greater precision, as well as to obtain genetically modified organisms, both prokaryotic and eukaryotic. The changes introduced by the CRISPR/Cas system are based on the repair paths of the single or double strand DNA breaks that cause insertions, deletions, or precise integrations of donor DNA. These changes are crucial for many fields of science, one of which is the use of animals (pigs) as a reservoir of tissues and organs for xenotransplantation into humans. Non-genetically modified animals cannot be used to save human life and health due to acute immunological reactions resulting from the phylogenetic distance of these two species. This review is intended to collect and summarize the advantages as well as achievements of the CRISPR/Cas system in pig-to-human xenotransplantation research. In addition, it demonstrates barriers and limitations that require careful evaluation before attempting to experiment with this technology.

## 1. Introduction

The increasing human life expectancy has led to an increase in the number of patients with chronic diseases and severe organ failure. Organ transplantation is an effective approach in the treatment of end-stage organ failure [1]. However, the imbalance between the supply of and the demand for human organs is a serious problem in contemporary transplantology. Since 2013, the rapid growth in interest in xenotransplantation has been related to the CRISPR/Cas technology appearance in genetic engineering [2]. In this review, we describe the CRISPR/Cas9 system, its use in genetic engineering, its advantages, and its limitations. We describe the contribution of this technology to the field of xenotransplantation research and justify its use for this purpose due to the existence of immunological and virological obstacles in pig-to-human xenotransplantation.

## 2. CRISPR/Cas Technology

### 2.1. Origin and Mechanism of Action

The CRISPR/Cas system has been described in bacteria and archaea as their acquired ‘immunity’ mechanism against viruses. It works by recognizing and hydrolyzing pathogen genetic material by means of CRISPR RNA (crRNA) associated with CRISPR-associated protein (Cas) nuclease [2,3]. The CRISPR/Cas system has been observed in 40% of bacteria and almost 90% of archaea whose genomes have been sequenced so far [4]. The CRISPR locus consists of a series of conserved repeating sequences interspersed with sequences called linker sequences. After entering the bacterial cell, the phage genome is hydrolyzed by Cas (Cas1 or Cas2) nuclease into small fragments of DNA, which are then inserted into the CRISPR locus of the host genome between linker sequences (as spacers). In response to a subsequent viral infection, spacer sequences are used as templates for the transcription of crRNA, which in combination with the trans-activating crRNA (tracrRNA) and the different Cas protein (e.g., Cas9 or Cas12) hydrolyze the target sequences of the genetic material of invading phages [2,5]. More than 40 different families of Cas proteins have been described. They are divided into three main types based on their sequences and structures, I, II, and III, and three others, IV, V, and VI. The presence of other types has also been confirmed. The CRISPR/Cas type II system requires only one Cas protein to function, Cas9, which contains the HNH nuclease domain and the RuvC nuclease-like domain, and therefore it has found application in genetic engineering. CRISPR/Cas9 has been shown to be a simple and efficient genome editing tool [2].

**Figure 1:**
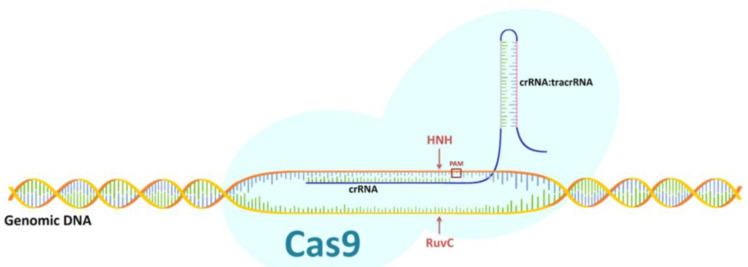
Diagram of CRISPR/Cas9 system. Description of a figure in the text.

The CRISPR/Cas9 system was first used in genetic engineering in 2013 [6]. Editing the genome through it depends on the formation of a double-strand break (DSB) and the subsequent cellular DNA repair processes [7]. One of these processes is repair by non-homologous end joining (NHEJ), and the other is homology directed repair (HDR), which is based on the homologous recombination mechanism that appears with the delivery of appropriately designed donor DNA [8]. In the bacterial CRISPR/Cas9 system, the mature crRNA binds to tracrRNA to form a tracrRNA:crRNA complex that partially binds to the Cas9 and guides the entire system to its target site [2]. The crRNA-specific hydrolysis of both DNA strands via CRISPR/Cas9 requires the presence of a complementary target sequence in the DNA, followed immediately by a protospacer adjacent motif (PAM) sequence. After the complementary connection of the crRNA molecule with the target site within one DNA strand, it is hydrolyzed by the Cas9 HNH nuclease domain, while the hydrolysis of the complementary DNA strand to the recognized target site is carried out by the Cas9 RuvC nuclease-like domain. This creates a two-strand DNA break three nucleotides downstream of the PAM sequence [9]. In genetic engineering, single-guide RNA (sgRNA) is used, which is the equivalent of the bacterial chimera: crRNA:tracrRNA (Figure 1).

In the sgRNA sequence, gRNA is distinguished as an element corresponding to the bacterial crRNA fragment that complements the target locus in DNA. The gRNA molecule is an element designed by the researcher [2]. Many variants of the CRISPR/Cas9 system have been developed in which the engineered gRNA recognizes a sequence from 18 to 24 nucleotides in length and Cas9 recognizes different PAM sequences from two to eight nucleotides in length. In genetic engineering, the system most commonly used is that found in *Streptococcus pyogenes*. It requires a 20-nucleotide gRNA molecule linked to a Cas9 nuclease and a three-nucleotide-long PAM sequence (specifically NGG, where N is an arbitrary base nucleotide followed by two guanosine nucleotides) [10].

### 2.2. Mechanisms of Repairing Double-Stranded DNA Breaks

Double-strand breaks generated by the CRISPR/Cas9 system may be repaired by DNA non-homologous end joining (NHEJ) or by homology directed repair (HDR). The HDR mechanism is based on homologous recombination, which takes place after delivery an appropriately designed donor DNA) [8,11].

NHEJ-mediated DNA repair generates small mutations such as insertions or deletions (indels) at the target sites. These mutations may interfere with/or abolish the function of target genes or genomic elements. It is known that the NHEJ process can occur by two pathways: classical (cNHEJ) and alternative (aNHEJ).

In mammals, the cNHEJ mechanism starts with the attachment of a DNA-dependent protein kinase (DNA-PK) complex to the DSB. In eukaryotes, the DNA-PK complex includes the Ku protein, composed of the two polypeptides, Ku70 and Ku80, as well as the DNA-dependent protein kinase (DNA-PKcs) catalytic subunit. The Ku protein is a molecular scaffold that enables the attachment of subsequent elements involved in the repair of the DNA double-strand breaks. In the next steps, the damaged or mismatched nucleotides are removed with Artemis nuclease. Then, the gap is filled with the λ polymerase or the μ polymerase. The final step is the ligation of the DNA ends with the DNA ligase IV complex consisting of the DNA ligase IV catalytic subunit and its cofactor [8,11,12]. The cNHEJ mechanism does not require the presence of microhomological sequences to work; however, when such fragments are present within the joined ends of the DNA, they can positively affect the connection of the DSB [13,14]. Moreover, it is known that cNHEJ is used more frequently when the cell is in the G0/G1 phase of mitosis [15].

The alternative HNEJ is also known as backup-NHEJ or microhomology-mediated end joining (MMEJ) [13]. In contrast to the cNHEJ, the alternative pathway often leads to large deletions, as this form of repair is most often based on microhomological sequences [16]. Therefore, in addition to large deletions, chromosomal translocations based on microhomological sequences are also observed [17,18]. This type of repair occurs mainly when the classic pathway is turned off or not fully functional (especially in the absence of Ku proteins) and relies on the polymerase θ [19,20].

Double-stranded DNA breaks can also be repaired by homologous recombination in the presence of an appropriately designed DNA construct. Homologous recombination takes place on a DNA template flanked with fragments complementary to the blunt ends of the DSB, called homologous arms [21]. Three models of homologous recombination are described: the Holliday model, the Meselson–Radding model (or the Aviemore model), and the Szostak model (or the double strand break model) [22]. Repairing HDR requires the presence of enzymes that allow DNA fragments to be joined together. A key role in this repair is played by the RAD51 protein, which mediates ATP-dependent DNA strand exchange. RAD51 enables quick searches of DNA fragments for homologous sequences and then facilitates strand exchange at the homology site. Homologous genome modification can be used to insert the desired genetic material, editing the genome of the target cell with high precision [23,24].

### 2.3. Difficulties and Limitations of Technology

The CRISPR/Cas9 system is not without limitations, mainly related to the formation of DNA breaks outside the target locus—off-target mutations—and dependence on the PAM sequence. Off-targets are one of the major concerns in genome editing via the CRISPR/Cas9 system. Compared to the use of zinc finger nuclease (ZFN) and TALE nuclease, the CRISPR/Cas9 system has a higher risk of developing off-target mutations in human cells [25]. This is related to genome size, as the larger the genome, the more DNA sequences that are identical or highly homologous to the target DNA sequences. In addition to target sequences, Cas9 nuclease linked to sgRNA hydrolyzes highly homologous DNA sequences, leading to mutations at undesirable sites [10,26,27]. Mutations outside the target site can lead to the dysfunction of some genes and sometimes even cell death. To ensure greater precision and specificity of the CRISPR/Cas9 system, it is necessary to select a target locus with as few potential off-target sites as possible at the bioinformatic analysis stage [28]. In addition, a modified Cas9—Cas9-D10A—nuclease with nickase activity was developed to minimize the formation of mutations in undesirable places. The Cas9-D10A in combination with sgRNA leads to the hydrolysis of one DNA strand at the target site. To obtain the hydrolysis of both DNA strands within a given target site, a system based on nickase Cas9-D10A and two sgRNAs should be designed, one of which is complementary to the coding strand and the other to the DNA template strand [29]. In this way, we can reduce the risk of mutations in undesirable places, while maintaining the precision of introducing modifications at the target locus. Another method is to use the truncated gRNAs containing 15 or fewer nucleotides. Shorter gRNAs have been shown to reduce mismatch tolerance and consequently reduce off-target frequency [30]; however, only sgRNA above 17 nt long efficiently led to the formation of changes at the target site [31]. It is also possible to use high-fidelity endonuclease variant of Cas9 protein, e.g., HypaCas9, which proved to be effective in modifying the target locus, while minimizing off-target effects [32]. It has also been shown that modifying standard SpCas9 to recognize an altered PAM sequence may reduce the amount of the off-target mutations [33]. Furthermore, the method of delivering the CRISPR/Cas9 system to the cells is of great importance. Delivery of the ribonucleoprotein (RNP) complex has been shown to minimize the off-target effect [34]. One of the new variants of the CRISPR/Cas9 method—prime editing—enables the most precise changes in the genome, limited to one base pair, minimizing the possibility of off-target mutations [35,36].

Theoretically, the CRISPR/Cas9 system can be applied to any DNA sequence using engineered gRNA. However, the specificity of the action of Cas9 nuclease depends on the 2–8 nucleotide PAM sequence located immediately downstream of the target sequence [2]. The identified PAM sequences differ among Cas9 protein orthologs (Table 1). The PAM sequence restricts the target site selection for the CRISPR/Cas9 system. The PAM sequences NGG and NAG occur in the genome on average once every eight nucleotides, while the PAM sequence NGGNG occurs once every 32 nucleotides, and NNAGAAW occurs once every 256 nucleotides. Therefore, the length of the PAM sequence affects the specificity of the CRISPR/Cas9 system. Mutations outside the target site for a system requiring a short PAM arise more frequently than when using a CRISPR/Cas9 system dependent on longer PAM sequences [10,37].

The existence of PAM sequence constraints is a major limiting factor in research using the CRISPR/Cas system. There are systems based on non-Cas9 nuclease. One of them is Cas12a (formerly known as Cpf1). It needs a single RNA molecule (crRNA) to function, not a crRNA:tracrRNA hybrid like Cas9 nuclease. Cas12a recognizes the T-rich PAM sequence, which is a great advantage as it could be used in places in the genome where it is not possible to design a cleavage with the standard Cas9 system. Moreover, it cleaves DNA via a staggered DNA DSB [45,46]. There is also a Cas12b nuclease, but its use is challenging due to the high temperature requirements [47].

It has also been proven that there are additional limiting factors in the use of the CRISPR/Cas system for genome modification. One of them is p53-dependent toxicity, which was noted in human pluripotent stem cells (hPSCs) after indel, obtaining efficiency greater than 80%. This process should be monitored when using the CRISPR/Cas9 system for genome modification [48]. In addition, repair of DSBs after CRISPR/Cas9 use may result in large inversions and deletions (about 10 kb), not just small indel mutations [49]. Repair may also result in the formation of chromosomal translocations [20] or in loss of one or both chromosomal arms [50]. Systems based on inactivated/dead Cas9 nuclease show lower genotoxicity due to lack of nuclease activity and non-formation of DSBs; however, their use depends on the purpose of the research, as their effects may be short-term. However, the CRISPR-STOP and iSTOP technology results in the permanent inactivation of the gene by introducing the stop codons into the modified sequence [51].

### 2.4. Limitations of CRISPR/Cas When Used in Large Animal Models

**Figure 2:**
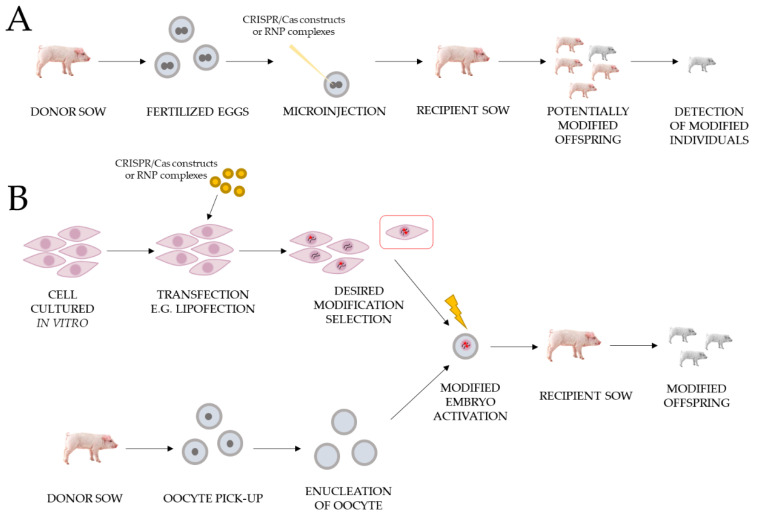
Workflows for obtaining gene edited pigs using the CRISPR/Cas system for xenotransplantation purposes. The figure shows two methods: (**A**) Microinjection of CRISPR/Cas modified constructs or RNPs complexes into porcine zygotes. (**B**) Somatic cell nuclear transfer (SCNT).

Large animal models could be obtained by microinjection of modifying constructs/RNPs of the CRISPR/Cas system or somatic cell nuclear transfer (SCNT) procedure. We detailed the advantages and disadvantages of these methods in our recent article [52]. Workflows of both methods are presented in Figure 2.

One of the biggest limitations of the CRISPR/Cas9 system’s use in large animal models is the occurrence of genetic mosaicism. CRISPR/Cas9 microinjection into zygotes often leads to individuals with more than two alleles for the targeted gene. This mosaicism in founder animals is the result of CRISPR/Cas9-mediated editing at different stages of embryo development. Overcoming mosaicism by breeding is possible in case of species with short generational intervals, but for large animals, it is not a viable solution. The timing of CRISPR/Cas9 components’ microinjection before the first round of DNA replication, the delivery method, and the short activity of Cas9 have an impact on the level of mosaicism. To reduce the mosaic mutations induced by the CRISPR/Cas9 system in the large animal model, it is proposed to provide CRISPR/Cas9 components into early pronuclear stage zygotes and use appropriate format Cas9-sgRNA (ribonucleoprotein complex) [53,54].

Another obstacle of the CRISPR/Cas9 technology is also the efficiency of HDR. HDR DNA repair takes place only during the S/G2 phase, when NHEJ may occur in all phases of the cell cycle. Therefore, it is believed that the NHEJ inhibition by chemical treatments should improve CRISPR/Cas9-mediated HDR. Moreover, the optimal design of the template DNA, and the choice of the proper type and length of donor DNA (ssDNA, dsDNA, or plasmid) can be crucial [53]. A further challenge of the CRISPR/Cas9 system is developing better delivery methods. The use of common adeno-associated virus (AAV) vectors is limited by the packaging capacity of foreign DNA and carrying sgRNA, Cas9, and donor template in a single AAV vector may be generally not possible. The problem can be solved by the use of smaller Cas9 orthologues [55] or the use of non-viral nanoparticle-based delivery of CRISPR/Cas9 strategies [56].

Despite the limitations associated with the use of the CRISPR/Cas9 system in genetic engineering, modifying the genome with it is very efficient, fast, and precise. The wide application of this system in various fields of science, including xenotransplantation, had a positive impact on their development.

## 3. Xenotransplantation

Xenotransplantation is described as any procedure that involves the transplantation, implantation, or infusion of the recipient (in this case, a human) cells, tissues, or animal organs. In addition, it also covers therapies that use human body fluids, tissues, organs, or cells that have come into contact ex vivo with animal organs, tissues, or cells. Xenotransplantation was first mentioned in 1667 in the context of the xenotransfusion of blood from a lamb to a human [57]. Many clinical trials of the use of animal organs have also been documented. One of them was a transplantation of a rabbit kidney to a human in 1905 [58]. Due to the phylogenetic proximity of non-human primates (NHPs) to humans, several studies were carried out in 1920–1990 using the kidneys, hearts, and livers of these animals [59]. As a result, however, the use of primates in xenotransplantation research was withdrawn due to the existing ethical concerns, the high risk of transmitting infectious diseases to humans, difficulties in reproducing these animals, and differences in organ sizes [60].

Since the 1990s, researchers have tried to use pigs as model animals in xenotransplantation research. The domestic pig (*Sus scrofa f. domestica*) is currently considered to be the most suitable candidate species. The reasons for selecting a pig as a donor animal include the relatively large litter size and short maturation period, the size and physiological similarity of its organs to human organs, and the low risk of xenozoonosis transmission [52,61]. However, there are discrepancies between pigs and humans which lead to the development of immune barriers that prevent a direct xenotransplantation. These differences lead to xenograft rejection [62]. Thanks to genetically modified pigs and immunosuppressive therapy, the survival outcomes of xenografts in primate recipients have improved significantly in preclinical xenotransplantation models.

### 3.1. Hyperacute Rejection (HAR)

Hyperacute rejection is the process that occurs minutes to hours after xenotransplantation. HAR is a type of humoral rejection mediated by antibodies that are naturally present in the recipient. The recipient antibodies binding to the epitopes present on the porcine endothelial cells causes the activation of the complement system. Activating the complement system leads to the lysis of the endothelial cells, which results in a destruction of the graft vascular system and in a subsequent failure of the transplant. This immune response is histologically characterized by a disturbance of the vessels’ integrity, edema, hemorrhage, and thrombosis with the deposition of antibodies and the end products of the complement system in the vessels [59,62,63,64].

The major xenoantigen involved in HAR is galactose-α1,3-galactose (α-Gal) [65]. This epitope is synthesized by the alpha-1,3-galactosyltransferase (GGTA1) enzyme encoded by the porcine *GGTA1* gene [66,67]. This enzyme catalyzes the galactose residue transfer reaction from galactose uridine diphosphate (UDP-Gal) to glycolipids and membrane glycoproteins through an α1,3 bond forming an α-Gal epitope on the cell surface. This enzyme is found in most mammals, including pigs, but not in humans and other primates. Therefore, in human blood, there are IgG antibodies directed against the α-Gal epitopes, which constitute as much as 1% of all circulating antibodies [68]. These natural antibodies are formed during the human neonatal period in response to the presence of gut bacteria that express the *GGTA1* gene [69].

Moreover, two more antigens have been described that significantly influence the occurrence of HAR. The first is cytidine monophospho-N-acetylneuraminic acid hydroxylase (CMAH), encoded by the porcine *CMAH* gene, responsible for the hydroxylation of N-acetyl neuraminic acid (Neu5Ac) to N-Glycolylneuraminic acid (Neu5Gc). Both of the above compounds belong to the sialic acid family and occur in the cell membrane glycoproteins. Neu5Gc antigen is not expressed in humans due to the presence of a mutation in the human *CMAH* gene, which disrupts its functionality [70]. Antibodies against porcine Neu5Gc antigen were proven to be present in human serum by in vitro experiments. The production of these antibodies can be induced after human consumption of pork meat [71]. The second antigen is beta-1,4-N-acetylgalactosaminyltransferase 2 (β4GalNT2) encoded by the porcine *β4GalNT2* gene, which is involved in the synthesis of the Sd(a) antigen—more precisely, the transfer of N-acetylgalactosamine (GalNAc) to galactose present in the alpha-2,3-sialic acid chains [72,73,74]. There is a gene encoding a homologous enzyme in the human genome; however, most people have low levels of anti-Sd(a) IgM antibodies that cause polyagglutination of red blood cells after blood transfusion from an individual with high expression of the human *β4GalNT2* gene and the presence of the Sd(a) antigen on the surface cells. Studies of the porcine *β4GalNT2* gene’s inactivation have also been performed and a reduction in the binding of human IgM and IgG antibodies to peripheral blood mononuclear cells of pigs has been demonstrated [75,76]. This suggests the presence of human antibodies which bind to the porcine antigen synthesized by the porcine β4GalNT2 enzyme.

It has been proven that the inactivation of the porcine *β4GalNT2* gene in combination with the inactivation of the porcine *GGTA1* and *CMAH* genes minimizes the activity of the β4GalNT2-dependent antibodies. Therefore, inactivation of porcine *GGTA1*, *CMAH* and *β4GalNT2* genes is a key in removing barriers related to HAR [75,77].

### 3.2. Acute Humoral Xenograft Rejection (AHXR)

Acute vascular rejection, also known as acute humoral xenograft rejection, occurs within days to weeks after xenotransplantation once HAR is under control [63]. It is a type of immune reaction that depends on antibodies, macrophages, and NK cells. Antibodies directed against swine leucocyte antigen (SLA) molecules play a dominant role in this response [77]. This type of xenograft rejection is similar to HAR, in that it originates in the vascular endothelium of small arteries. This response begins with the binding of the recipient’s antibodies to antigens on the surface of the donor’s cells. The generated antibody–antigen complex leads to the adhesion of NK cells and macrophages, which then invade the interstitial space of the transplanted organ [78]. These cells produce cytokines such as tumor necrosis factor α (TNFα) and interferon γ molecules [79]. The cascade that affects the expression of genes encoding adhesion and chemotactic factors is triggered, which in turn lead to aggregation and adhesion of the recipient’s platelets. The formation of clots in the lumen of small arteries directly causes the acute vascular rejection [80].

In the xenotransplantation with the domestic pig as a model organism, it is possible to modify the class I swine major histocompatibility complex (swine leucocytes antigens, SLA) molecules. For this purpose, the beta-2-microglobulin (β2M) activity encoded by the pig *β2M* gene was disabled. It has been proven that the skin transplanted from such genetically modified pigs caused a delayed immune response compared to the control unmodified tissues [81].

To reduce the cytotoxicity of recipient NK cells, pigs expressing human leukocyte antigen E (HLA-E) and human CD46 (cluster by differentiation 46), also known as membrane cofactor protein (MCP) were prepared. The presence of both transgenes in porcine cells has been shown to reduce the cytotoxic response of NK cells [82,83]. The direct cytotoxicity of NK cells is tightly regulated by the balance between activating and inhibiting signaling pathways. They are mediated by activating receptors for NK cells [84]. Porcine UL16 binding protein 1 (ULBP1) encoded by the *ULBP1* gene has been shown to be present on porcine vascular endothelial cells [85]. This protein is a functional ligand for the natural killer cell lectin like receptor K1 (NKG2D) activating receptor of human cells. Binding of the porcine ULBP1 protein to the human NKG2D receptor results in the release of the lytic granules that mediate the destruction of xenograft cells [86,87]. In addition, immunoglobulin-like receptors that inhibit the cytotoxicity of NK cells present on their surface poorly recognize class I SLA molecules and, consequently, turn off signals that inhibit NK cell activation. These receptors include: killer-cell immunoglobulin-like receptor (KIR) and immunoglobulin-like transcript 2 receptor (ILT2), as well as the family of the C lectin receptors: cluster of differentiation 94/natural killer cell lectin like receptor K1 (CD94/NKG2A) [88,89]. Elimination of the porcine ligands for NK cell-activating receptors coupled with an increase in the expression of the ligands for inhibitory receptors may prevent NK cell-mediated xenograft rejection. In one experiment, animals expressing the human *CD55* gene, also known as the decay-accelerating factor (DAF), and with inactivated porcine *ULBP1* gene were obtained. Cells from genetically modified and unmodified pigs were incubated with human serum. It was shown that the viability of genetically modified cells was 84.4% on average, and 43.8% of unmodified cells [90].

Complement inhibition has also been achieved by producing transgenic pigs expressing human CD55 and CD59 known as membrane reactive lysis inhibitor (MIRL). The presence of the CD55 and CD59 protein factor on the surface of human vascular endothelial cells protects them from the negative influence of the complement system. The appearance of these proteins on the surface of porcine cells could help to protect against the development of a negative immune response after xenotransplantation. In in vitro studies, the double transgenic (hCD55 and hCD59) and unmodified porcine fibroblasts were incubated with human serum. The viability of genetically modified cells was achieved at the level of 77.3%, while for control cells the survival was close to 0% [91].

Macrophages exert a direct toxic effect on xenograft cells mediated by the production of pro-inflammatory cytokines TNFα, interleukin-1 (IL-1) and interleukin-6 (IL-6). Human signal-regulatory protein α (SIRPα) present on the surface of the macrophage cell membrane acts as an inhibitory receptor. Human SIRPα interacts with its ligand, CD47 (cluster of differentiation 47), also known as integrin associated protein (IAP), found on the surface of cells. Recognition of CD47 by SIRPα prevents macrophage-mediated autologous phagocytosis [92,93]. The amino acid sequence similarity of porcine and human CD47 proteins is 73%. Interspecies incompatibilities lead to the disruption of the porcine CD47 full functionality in the human body, resulting in macrophage phagocytosis of xenograft cells. The role of macrophages in xenograft rejection can be overcome by expressing the human *CD47* gene in pigs. The presence of the CD47 molecule on the surface of porcine cells inhibits the phagocytosis of these cells by macrophages [93].

### 3.3. Acute Cellular Rejection (ACR)

Acute cellular rejection is the next stage of the immune response that appears several days after transplantation [94]. In the case of xenografts (in the pig-to-primate monkey system), this type of rejection is very rarely recorded. It is associated with the onset of a humoral reaction that is faster and more violent. In recent years, multitransgenic animals have been obtained, reducing the problem of HAR and AHXR, and therefore more and more attention is being paid to the type of cellular rejection [78]. The cellular immune response is based on T and B lymphocytes, macrophages and, in part, NK cells. However, the main role is played by CD8 + and CD4 + T cells, which interact with class I and II SLA molecules of the donor [95]. The next stage involves the infiltration of these cells into the interstitial space of the xenograft and the formation of necrotic foci within the tissues.

The T lymphocytes’ activation requires the T-cell receptor (TCR) binding to major histocompatibility complex (MHC) molecules on antigen-presenting cells (APCs). Compatibility of MHC molecules between species is a key issue in the context of xenotransplantation [96]. To counteract the T cell response, transgenic pigs expressing cytotoxic T cell antigen 4 (CTLA-4) were produced. This protein is responsible for the inhibition of the T lymphocytes activity. It has been shown that due to the high and constitutive expression of the CTLA-4 antigen, the transgenic individuals were immunologically impaired, which resulted in their premature death [97,98]. Subsequent experiments focus on obtaining animals expressing CTLA-4 antigen in selected tissues. Another approach is the expression of the human dominant-negative mutant class II transactivator (CIITA-DN) transgene—a transactivator of human class II major histocompatibility complex molecules in porcine cells. This modification reduces the expression of class II SLA molecules and inhibits the human T cell response to porcine xenograft [99]. It is also possible to switch off the expression of class I SLA molecules [100]. Both applied modifications showed a reduced porcine antigens presentation on APC cells. However, the effect of the above modifications requires verification in preclinical studies with the use of organ xenotransplantation between pigs and primates.

To obtain the desired effect in xenotransplantation, it is necessary to combine modifications preventing hyperacute, vascular, and cellular rejection, which became possible thanks to the CRISPR/Cas system.

### 3.4. Coagulation System Dysregulation

Dysregulation of the recipient’s coagulation system constitutes one of the main barriers in xenotransplantation. They appear in parallel with HAR and AHXR [65]. Dysregulation of the coagulation system causes the thrombotic microangiopathy development in the xenograft [101]. Features of thrombotic microangiopathy include fibrin deposition and platelet aggregation, resulting in thrombosis within the blood vessels of the graft and ultimately ischemic damage [102]. As coagulation disorders develop, the recipient often has systemic consumption coagulopathy, which may lead to his death [103]. Coagulation is a complex process that also involves interactions with factors related to inflammation and innate immunity. It usually occurs continuously in the bloodstream, but is limited by anticoagulants that maintain its homeostasis [104]. When endothelial cells are damaged, tissue factor (TF) activates the external clotting pathway [105]. Increased coagulation is initiated by TF which complexes with factor VIIa. The coagulation cascade is then enhanced by the emerging factors (complex VIIa/TF, IXa and Xa), which in turn activate thrombin [106]. Inhibitory pathways that regulate the coagulation balance are triggered, including the tissue factor pathway inhibitor (TFPI) and the thrombomodulin (TM)-protein C (PC) pathways [107].

In the xenotransplantation, antibody attack on xenograft cells and activation of the complement system transform porcine endothelial cells from an anticoagulant phenotype into a procoagulant state [108]. Tissue factors from both the recipient and the donor contribute to the activation of the external coagulation cascade [109,110]. Molecular incompatibilities between the coagulation systems and its inhibition in primates and pigs amplify this process, leading to the destruction of the xenograft blood vessels, as well as tissue infiltration by various immune cells. Porcine TFPI is not sufficient to inhibit factor Xa in primates and fails to effectively turn off the primary recipient TF [110,111]. The porcine thrombomodulin-protein C pathway also fails to regulate the thrombin pathway in primates. Porcine thrombomodulin binds to human thrombin to a lesser extent due to structural differences in the molecules. Thus, it does not properly activate protein C [112]. Another molecular incompatibility occurs between porcine von Willebrand factor (vWF) and platelet glycoprotein 1b (GP1BB) beta polypeptide of primates [113]. Porcine vWF spontaneously aggregates blood platelets from non-human primates via GP1BB receptors. After abnormal interaction between GP1BB-vWF molecules, activation of platelets occurs, leading to the development of thrombosis. This takes place after the recruitment of platelets to the site of endothelial cell damage, which results in the widespread activation of the coagulation system [114,115].

Recent advances in xenotransplantation have resulted in a better understanding of the immune mechanisms, including those related to the clotting system, that underlie the failure of swine xenografts. Studies related to the expression of human thrombomodulin in pigs have been performed. Transgenic porcine aortic endothelial cells (with human TM) have been shown to significantly inhibit prothrombinase activity and delay human blood clotting [116,117]. They also show less activity in inducing platelet aggregation of the recipient. In clinical trials, the expression of human TM in the pig allowed for an increase in the survival time of a heart xenograft in the baboon [118,119]. Another very important factor is the endothelial cell protein C receptor (EPCR). It mediates anti-inflammatory, anticoagulant, and cytoprotective signaling [120]. The in vitro studies have shown that cells from transgenic pigs expressing human CD46 and EPCR (and with *GGTA1* gene knock-out) reduce the activity of human platelet aggregation [116]. Another approach is to try to address the incompatibility problem between porcine and human tissue factor inhibitor. Another in vitro study showed that expression of human TFPI on the surface of porcine cells inhibits the activity of the human tissue factor [121]. In addition, the enzyme known as CD39 (cluster of differentiation 39), or ectonucleoside triphosphate diphosphohydrolase-1 (NTPDase 1), encoded by the *ENTPD1* gene, may play a key role in the regulation of the coagulation system. This enzyme is responsible for catalysis of the extracellular adenosine triphosphate (ATP), adenosine diphosphate (ADP) and adenosine monophosphate (AMP) degradation reactions. Its role in the regulation of coagulation is to inhibit the formation of clots. Expression of the human CD39 enzyme in a transgenic pig has been shown to protect against myocardial ischemia and reperfusion injury [122]. There are also factors that are expressed in individual organs that pose a problem in xenotransplantation. One of them is von Willebrand factor, which is involved in the pathogenesis of transplant failure in lung xenotransplantation [123]. To suppress this response, the porcine *vWF* gene was inactivated. After lung transplantation from such genetically modified pigs to primates, a significant decrease in platelet aggregation of the recipient was noted [124]. Lethal thrombocytopenia accompanying liver xenotransplantation is another barrier resulting from differences in the function of the coagulation system. Human platelets are bound by the asialoglycoprotein receptor (ASGR) encoded by the *ASGR1* and *ASGR2* genes. They are expressed in liver sinusoidal endothelial cells (LSEC). It has been proven that this receptor binds human platelets and causes their phagocytosis. Human platelet sequestration causes thrombocytopenia and xenograft rejection. It has been reported that inactivation of the porcine *ASGR1* gene coding the first subunit of the asialoglycoprotein receptor in pigs may prevent this reaction. Livers from genetically modified pigs show reduced platelet uptake of humans in in vitro perfusion studies [125,126].

### 3.5. Preclinical Studies

With the development of genetic engineering that makes it possible to obtain pigs with various genetic modifications and more effective immunosuppressive methods, there have been many promising preclinical advances in recent years. Old World non-human primates, due to their high immunological similarity to humans, are commonly used as surrogates in experimental xenotransplantation model. The most important achievements in preclinical xenotransplantation studies are presented below. Genetically modified pig kidney has the potential to be the first clinically applied pig-to-human organ transplants [127]. In recent years, significant progress has been made towards extending the survival time of life-supporting porcine kidney xenografts in non-human primates, with the longest survival time being 499 days [128]. The authors used kidneys derived from genetically modified pigs with knock-out of porcine *GGTA1* gene and knock-in of human *CD55* gene, and applied immunosuppression included a transient pan-T cell depletion and an anti-CD154-based regimen combined with daily mycophenolate mofetil (MMF) and solumedrol. The studies suggested that CD4+ T cell depletion may play a crucial role in the long-term survival. For cardiac xenotransplantation, the results are even more promising. Heterotopic hearts from multimodified pigs with knock-out of porcine *GGTA1* and knock-in of human *CD46* and *TM*, combined with anti-CD40 antibody based treatment, survived up to 945 days in the baboon [118]. Exploiting the same genetic background and immunosuppression regimen, orthotopic cardiac xenotransplantation was performed with a maximum survival of 195 days [83]. Porcine liver and lung xenotransplantations are more problematic than porcine kidney and heart due to severe coagulation dysregulation. Orthotopic liver from pigs with knock-out of porcine *GGTA1* and under immunosuppressive therapy and exogenous human coagulation factors survived for 29 days in the baboon [129]. In turn, prolonged survival time for orthotopic lungs xenografts (31 days in immunosuppressed baboon recipient) was achieved using multimodified pigs with knock-out of porcine *GGTA1* and *β4GalNT2* genes and knock-in of human *CD46*, *CD47*, *TM*, *EPCR*, *HO1* genes [130].

### 3.6. Virological Concerns

The viruses that pose the greatest threat in xenotransplantation are porcine endogenous retroviruses. These pathogens are integrated into the pig genome. In the porcine genome, the PERV provirus has been recorded with a copy number ranging from 1 to 100 [131]. PERV viruses are classified into three subtypes: PERV-A, PERV-B, and PERV-C [132]. Type A and B occur in the genome of all porcine breeds, in contrast to type C, the presence of which is reduced [133]. The transfer of the recombinant PERV-A/C virus into human cells in in vitro culture was observed. This phenomenon has not been observed in vivo [134]. In the context of xenotransplantation, the PERV proviral genes should be eliminated from the porcine genome, as recommended by the International Xenotransplantation Association [135].

The other viruses that pose a risk in the context of pig-to-human xenotransplantation include porcine cytomegalovirus (PCMV), porcine lymphotropic herpesvirus (PLHV) and hepatitis E virus (E-HEV) [136,137,138]. However, there are effective methods for eliminating all the above viruses by maintaining appropriate conditions for animals and regular veterinary examinations.

### 3.7. Legal, Social and Ethical Concerns

Despite the many advantages of xenotransplantation, there are some ethical, legal, and social aspects that must be regulated and considered. First, there are no international legal regulations for pig-to-human xenotransplantation [139]. Their establishment is one of the major milestones in the introduction of pig-to-human xenotransplantation. Apart from the lack of legal regulations, introducing xenotransplantation into clinical practice requires a discussion in the society combined with explanation of procedures and knowledge bases. There are concerns that some societies will oppose pig-to-human xenotransplantation because of religious and cultural aspects [140]. Moreover, there is a probability that the use of organs from modified pigs, in some countries, may lead to a global trend of reducing the number of human transplants for altruistic reasons [141]. At this moment, we are unable to predict the reaction of society to a person carrying an animal organ. The social acceptance and the fear of social rejection may pose a risk of a medical procedure refusal by patient. Therefore, there is a need for xenograft recipient anonymity.

The ethical aspects of xenotransplantation mainly concern the use of animals, namely pigs. Harvesting organs from these animals and transplanting them into humans will in most cases result in the death of the animal. Moreover, another aspect concerns the welfare of animals bred for xenotransplantation. These animals are likely to be kept in sterile, laboratory-like conditions. From a human standpoint, the level of care provided to these animals will be the highest. However, when we change to the animal perspective, these conditions may be worse than in agricultural breeding. This type of approach is anthropocentric and puts human life above that of other animal species [142]. For this reason, it is imperative to investigate all alternatives that could replace animal organs and save patients’ lives before introducing pig-to-human xenotransplantation into clinical practice [143]. These alternatives so far include, but are not limited to, the use of stem cells and tissue engineering [144]. Thus far, no alternative has been found that would be able to fully replace the functionality of the human organ. For this reason, pig-to-human xenotransplantation is still considered to rescue patients with extreme organ failure.

## 4. Pigs Modified with CRISPR/Cas Technology in Xenotransplantation

The first pigs modified against HAR (using the CRISPR/Cas9 system) with a triple knock-out of *GGTA1*, *CMAH*, and *β4GalNT2* was obtained in 2015. A triple knock-out of these porcine genes was proven to minimize the binding of baboon and chimpanzee IgM and IgG to porcine peripheral blood mononuclear cells (PBMC) by more than 90% [75]. In contrast, the first pigs modified with the CRISPR/Cas9 system showed a knock-out of class I SLA molecules. Two individuals showing the desired changes indicated a lower level of T CD8+ lymphocytes [100]. Another triple knock-out of the pig genes *GGTA1*, *CMAH* and *iGb3S* was obtained in five animals, where it was shown that cells from modified pigs indicated a statistically significantly lower binding of human IgG and IgM [125]. However, one year later it was proved that the porcine *iGb3S* gene product did not affect the level of porcine cells surface HAR-causing epitopes, thus it was eliminated from modifying for xenotransplantation purposes [145]. Multiple research groups have received single, double or triple knock-out of porcine *GGTA1*, *CMAH* and *β4GalNT2* genes in various combinations [146,147,148,149,150,151,152,153,154]. In each of these studies, the usefulness of these modifications was proved, and it was concluded that they are necessary to study the effects of subsequent modifications. Combined with the porcine *GGTA1* and *CMAH* genes knock-out by CRISPR/Cas9 technology, with a simultaneous knock-in of the human *CD55* gene with the expressing cassette was successfully obtained [155]. In 2016, using the CRISPR/Cas system, multi-transgenic pigs with knock-in of human *CD46*, *CD55*, *CD59*, *A20* (cDNA cassette) and *HO1* (cDNA cassette) genes were obtained using the previously obtained double knock-out carrier of porcine *GGTA1* and *CMAH* genes [79]. These multi-mods were selected for their comprehensive HAR and AHXR prevention. Using the second-generation nuclease FokI-dCas9, a precise knock-in of the human gene encoding the anti-CD2 mAb was obtained in exon 9 of the porcine *GGTA1* gene, causing its simultaneous inactivation [156]. A *GGTA1*/SLA class I double knock-out was also obtained, proving the reduced affinity of porcine PMBC for human IgG and IgM [153]. In 2018, by means of the nickase Cas9-D10A, pigs with inactivated porcine *ULBP1* gene were obtained. The primary porcine aortic endothelial cells (PAEC) have been shown to be less susceptible to killing by human NK cells [85]. Another group obtained modified individuals with triple knock-out of the porcine *GGTA1*, *β2M*, and *CIITA* genes by CRISPR/Cas9 technology. They proved that porcine modified PBMCs did not effectively activate human CD4 + and CD8 + T cells; moreover, skin grafts from modified pig survived in immunocompetent mice longer than the wild type control grafts [157]. The most comprehensive genetic changes by CRISPR/Cas9 in porcine genome for xenotransplantation purposes were obtained in 2020, when individuals with quadruple knock-out of pig *GGTA1*, *CMAH*, *β4GalNT2*, and *β2M* genes and a knock-in of five human genes: *CD46*, *CD55*, *CD59*, *HO1* and *A20* were obtained. They performed human complement activation tests in vitro, which showed significantly lower affinity of human IgG/IgM to porcine kidney cells [77]. Recently, multitransgenic animals with inactivated porcine genes—*GGTA1*, *CMAH* and *β4GalNT2*—and with the expression of nine transgenes—hCD46, hCD55, hCD59, hB2M, HLA-E, hCD47, hTHBD, hTFPI and hCD39–have been obtained. Moreover, 25 copies of PERV were inactivated in the same individuals [158]. These animals may be used for preclinical studies with NHPs and for further experiments with additional changes in the porcine genome for xenotransplantation.

Modifications (insertions, deletions, transitions) after using the CRISPR/Cas system to obtain genetically modified pigs useful for xenotransplantation purposes are summarized in Table 2. Although most modifications are small indel mutations, there have been both larger insertions and deletions after application of this genetic engineering technology.

One of the most promising achievements in pig-to-human xenotransplantation research beyond the elimination of immune barriers was the achievement of porcine endogenous retroviruses’ (PERVs) inactivation and the insertion of porcine *RSAD2* gene into porcine *Rosa26* locus. The PERVs were inactivated for the first time in 2015 using the CRISPR/Cas9 system. In the experiments, all 62 copies of the virus present in one individual were excluded. For this purpose, a gRNA complementary to the *pol* gene encoding the polymerase present in the sequences of each PERV type was designed. This gene codes a protein that acts as reverse transcriptase and is necessary for the correct replication of the virus genetic material and its infection [159]. The porcine kidney epithelial cell line (PK15) was tested after inactivation of the PERV proviral genes and the formation of impaired non-infectious viral particles was observed. The genetically modified PK15 cells were protected against reinfection with PERV, while the performed 55-day observations do not provide certainty as to the durability of the obtained changes [160]. Another group received PERV-inactivated primary porcine cell lines by combining CRISPR/Cas9 technology with a p53 inhibitor, pifithrin-α (PFTα), and a growth factor, bFGF. Subsequently, they obtained PERV-inactivated pigs through somatic cell nuclear transfer (SCNT) [161].

To counteract viral threats in pig-to-human xenotransplantation, the porcine *RSAD2* gene was inserted into porcine *Rosa26* locus by CRISPR/Cas9 in 2020. The RSAD2 protein, known as viperin, exhibits antiviral activity (by, e.g., promoting dendritic cells to interferon beta production) against different viruses such as: zika, hepatitis C, HIV, influenza A, but the most important for xenotransplantation purposes classic swine fever virus (CSFV) and pseudorabies virus (PRV). This knock-in was performed into the porcine *Rosa26* locus enabling the transgene constitutive expression. The results show the reduced infection of cells by CSFV and PRV [162].

## 5. Conclusions

The use of the CRISPR/Cas system in genetic engineering has enabled the enormous development of pig-to-human xenotransplantation research. Thanks to this technology, animals with multi-modifications were obtained, enabling the reduction in the primates’ immune reaction to a porcine xenograft. Most of the genetic changes were obtained with the natural DNA repair system—NHEJ—after the formation of DSBs in DNA. The main mutations were small insertions and deletions, while insertions above 200 bp and deletions above 100 bp were also obtained. The big challenge is to prevent off-target mutations. The unpredictability of changes and off-target mutation formation prompts researchers to look for new solutions, most often related to the modification of the basic CRISPR/Cas9 system, e.g., nickase Cas9-D10A, prime editing.

In addition to efforts to control the primates’ immune response in xenotransplantation research, the virological aspect is of great importance. However, it has been proven that this obstacle can be successfully removed using CRISPR/Cas9 technology. So far, successful removal of the problem of viruses such as PERV, CSFV and PRV has been described.

Although it is not yet possible to obtain pig organs for human transplantation, the CRISPR/Cas system brings researchers closer to this goal. The aspects described in this review show that the application of the CRISPR/Cas9 system in xenotransplantation research has enabled dynamic development in this field. A variety of ways to use this precise genome modifying technique, while minimizing the problem of mutation formation outside the target (e.g., delivering RNP complexes) enables the selection of an appropriate CRISPR workflow to produce modified pigs. Therefore, the greatest challenge remains to establish and obtain an appropriate combination of porcine genome multimodification to counteract the human immune response to pig organs and to ensure virological safety. As research into the “ideal” modified pig is ongoing, it seems like a matter of time before researchers obtain one thanks to CRISPR/Cas technology. Success in xenotransplantation research will have one major advantage over human-to-human transplantation—the donation of pig organs will not be limited, which will reduce the number of patients on the waiting list for transplantation.

## Figures and Tables

**Table 1:** Examples of Cas9 protein orthologs.

Ortholog	Host Organism	PAM Sequence (5′→3′)	References
NmeCas9	*Neisseria meningitidis*	NNNNG(A/C)TT	[38]
SaCas9	*Staphylococcus aureus*	NNGRRT or NNNRRT	[39]
St1Cas9	*Streptococcus thermophilus*	NNAGAA or NNAGAAW	[40]
ScCas9	*Streptococcus canis*	NNG	[41]
CjCas9	*Campylobacter jejuni*	NNNNACA	[42]
FnCas9	*Francisella novicida*	YG	[43]
St2Cas9	*Streptococcus thermophilus*	NGGNG	[44]

**Table 2:** Summary of genetic changes introduced by the CRISPR/Cas system into the porcine genome for xenotransplantation purposes.

Modification	Number ^1^	Occurred Repair			References
		Alleles	Mutation Type	Length (bp)	
*GGTA1*-KO	1	1 2	deletion deletion	−5 −7	[75]
*CMAH*-KO	1	1 2	deletion deletion & insertion	−12 −3/+5	
*β4GalNT2*-KO	1	1 2 3 *	insertion deletion deletion	+1 −5 −12	
SLA class I KO	1	1 2	deletion deletion	−276 −276	[100]
	2	1 2	deletion deletion	−276 −4	
*GGTA1*-KO	1	nd ^#^	insertion	+1	[125]
	2	nd ^#^	deletion	−4	
	3	nd ^#^	deletion	−7	
	4	nd ^#^	insertion	+1	
	5	nd ^#^	insertion	+1	
*CMAH*-KO	1	nd ^#^	deletion	−5	
	2	nd ^#^	insertion	+1	
	3	nd ^#^	deletion	−5	
	4	nd ^#^	deletion	−15	
	5	nd ^#^	insertion	+1	
*iGb3S*-KO	1	1	deletion	−2	
		2	deletion	−72	
	2	1	deletion	−15	
		2	insertion	+1	
	3	nd ^#^	insertion	+1	
*GGTA1*-KO	1	nd ^#^	insertion	+1	[149]
*CMAH*-KO	1	nd ^#^	deletion	−15	
*GGTA1*-KO	1	1	insertion	+1	[151]
		2	insertion	+421	
*CMAH*-KO	1	1	deletion	−103	
		2	deletion	−103	
*GGTA1*-KO	1	1	insertion	+1	[150]
		2	insertion	+1	
	2	1	insertion	+1	
		2	none	−	
		3	insertion	+2	
	3	1	deletion	−12	
		2	deletion	−3	
		3	none	−	
	4	1	deletion & insertion	−10/+4	
		2	insertion	+1	
	5	1	deletion & insertion	−1/+3	
		2	deletion	−4	
*GGTA1*-KO	1	nd ^#^	insertion	+1	[146]
*CMAH*-KO	1	nd ^#^	insertion	+1	
*β4GalNT2*-KO	1	nd ^#^	deletion	−10	
*ULBP1*-KO	1	1	deletion & insertion	−21/+2	[85]
		2	deletion & insertion	−4/+13	
	2	1	deletion	−40	
		2	deletion	−29	
	3	1	deletion & insertion	−21/+1	
		2	deletion	−27	
	4	1	deletion	−29	
		2	deletion	−34	
	5	1	deletion	−29	
		2	deletion & insertion	−18/+2	
*GGTA1*-KO	1	1, 2	deletion	−271	[157]
*β2M*-KO	1	1	double deletion	−2/−11	
		2	deletion & double insertion	−6/+1, +27	
*CIITA*-KO	1	1, 2	double deletion & insertion	−5, −6/+4	
*GGTA1*-KO	1	1, 2	deletion	−11	[77]
*CMAH*-KO	1	1	insertion	+1	
		2	deletion	−3	
*β4GalNT2*-KO	1	1	deletion	−5	
		2	insertion	+367	
*β2M*-KO	1	1	deletion & transversion	−2/T→G	
		2	deletion	−53	
		3 *	insertion	+279	
*GGTA1*-KO	1	1	deletion	−10	[158]
		2	vector insertion	−	
*CMAH*-KO	1	1	deletion	−391	
		2	insertion	+2	
*β4GalNT2*-KO	1	1	deletion	−13	
		2	deletion	−13	
		3	deletion	−14	
		4	deletion	−14	

^1^ The number of the genetically modified individual; * Gene duplication; ^#^ nd—no data.

## Data Availability

No new data were created or analyzed in this study. Data sharing is not applicable to this article.
